# Fermentation-synergized physical modification: Enhancing physicochemical properties and bioactivities of soluble dietary fiber from peanut shells

**DOI:** 10.1016/j.fochx.2025.102667

**Published:** 2025-06-16

**Authors:** Qiong Wu, Xinru Wu, Zifei Wang, Zhitong Cai, Yonggang Dai

**Affiliations:** a Changchun University, Nanguan District, Changchun, Jilin Province 130022, China; b Jilin Academy of Agricultural Sciences, Nanguan District, Changchun, Jilin Province 130033, China

**Keywords:** Peanut Shell, Soluble dietary Fiber, Physical modification, Biological activity

## Abstract

In this study, *Lactiplantibacillus plantarum* BNCC 339790 was employed to extract soluble dietary fiber (SDF) from peanut shells, followed by modification using ultrasonic, microwave, and ultra-high-pressure methods to investigate their effects on SDF properties. Ultra-high-pressure modification increased SDF's water-holding and oil-holding capacities by 1.74-fold and 2.07-fold, respectively. Characterization revealed decreased crystallinity and altered surface morphology with increased roughness and porosity after modification by the three methods. In addition, microwave-treated SDF exhibited better thermal stability. Ultrasonic treatment significantly enhanced SDF's inhibitory effects on pancreatic lipase (from 62.44 % to 83.33 %) and α-amylase (from 79.81 % to 94.94 %), demonstrated improved hypoglycemic and hypolipidemic potential. Furthermore, ultrasonically modified SDF exhibited significant anti-inflammatory activity by significantly suppressing TNF-α, IL-6, and IL-1β expression. This study provides a foundation for the further development of SDF and its promotion in functional foods and other potential applications.

## Highlights

• Soluble dietary fiber is prepared by fermentation-synergized physical modification.
• Modified soluble dietary fiber shows enhanced physicochemical characteristics.
• Superior hypoglycemic activities are exhibited by modified soluble dietary fiber.
• Stronger anti-inflammatory activity is exhibited by modified soluble dietary fiber.

## 1 Introduction

Peanuts are widely cultivated in Asia and America. China, as a major peanut-producing country, plays a crucial role in peanut production, consumption, and export trade. As a by-product of peanut processing, peanut shells (PS) have an annual production of approximately 7.44 million tons, only a small amount is used as feed or fuel, while the majority is discarded or directly incinerated, causing serious environmental pollution and a huge waste of biomass resources (Li et al., 2023; Oulidi et al., 2022). It is abundant in nutrients, including protein (4.8 % - 7.2 %), crude fat (1.2 % - 1.8 %), minerals (e.g., Na, K, Ca, Mg), and bioactive substances such as flavonoids and saponins (Wang et al., 2023). As it is enriched with crude fiber (65.7 % - 79.3 %) and hemicellulose (10.1 % - 11.6 %), it can be considered a premium source of dietary fiber (DF) (Fan et al., 2024), making PS valuable for applications in feed, fuel, adsorbents, construction, and other fields.

DF primarily derives from plant materials and consists of non-starchy carbohydrates and lignin (Zhang et al., 2024). It can be categorized into soluble dietary fiber (SDF) and insoluble dietary fiber (IDF). SDF helps regulate blood sugar levels, protect the cardiovascular system, promote intestinal peristalsis, and enhance antioxidant and anti-inflammatory capabilities (Guan et al., 2021; Wu et al., 2022), thereby attracting widespread attention and in-depth research from scientific researchers and professionals in the food and pharmaceutical industries. Natural SDF has issues such as low solubility, excessive viscosity (caused by high pectin content), and insufficient biological activity, making it difficult to meet market demand for complex formulations. Therefore, there is an urgent need to modify the extracted SDF to break through its natural structural limitations, thereby specifically enhancing its functional activity, improving its processing characteristics, and expanding its range of applications.

SDF modification can be achieved through physical, chemical, or biological approaches. Chemical modification (e.g., acid-base treatment, esterification, phosphorylation, and acetylation) may introduce new chemical groups and residues, often raising concerns about chemical residues and food safety risks (Yang et al., 2022). Biological modification (e.g., microbial fermentation and enzymatic treatment) faces challenges in scalability due to strict environmental requirements (Guo et al., 2025; Liu et al., 2024). In contrast, physical methods (e.g., mechanical grinding, heat, ultrasonic, and microwave  treatments) offer advantages in safety, cost-effectiveness, and industrial feasibility (Ji et al., 2024; Yu et al., 2025). For instance, Zhao et al. (2024) modified soybean dregs SDF with steam blasting, and the modified SDF exhibited significantly enhanced structural characteristics and emulsification properties compared to the unmodified counterpart. Dong et al. (2022) showed that the physically modified SDF structure showed significant roughening while its water retention and cholesterol adsorption capacity were significantly enhanced. The research results all confirmed the effectiveness of the modification strategy.

Current research on SDF from PS mainly concentrates on improving extraction techniques and analyzing physicochemical properties (Dong et al., 2020; Fan et al., 2024; Li et al., 2022; Wang et al., 2024). However, systematic studies on the impact of modification techniques on its functional properties remain limited. To address this gap, our study presents the first comprehensive investigation of fermentation-assisted physical modification (including ultrasonic, microwave, and ultra-high-pressure treatments) on the properties of SDF. This work not only advances the understanding of SDF modification strategies but also provides a foundation for the high-value utilization of PS in food and industrial applications.

## 2 Materials and methods

### 2.1 Materials and reagents

PS (Peanut Research Institute of Jilin Academy of Agricultural Sciences, Changchun, China), the yellow-brown shells without mechanical damage and mold were selected, oven-dried at 55 °C until constant weight, and then crushed through a 60-mesh sieve. High-temperature resistant α-amylase (20,000 U/mL), saccharase (100,000 U/mL), α-glucosidase (50 U/mg), and *p*-nitrophenyl-α-D-glucopyranoside (PNPG) (Shanghai Yuanye Biologicals Co., Ltd., Shanghai, China). Neutral protease (800 U/mg), acarbose, α-amylase (600 U/mL), pancreatic lipase, and *p*-nitrophenyl palmitate (PNPP) (Aladdin Biochemistry & Technology Co., Ltd., Shanghai, China). CCK8 (Dalian Meilun Biotechnology Co., Ltd., Dalian, China). ELISA kit (Enzyme Labelling Biotechnology Co., Ltd., Yancheng, China). *Lactiplantibacillus plantarum* BNCC 339790 (BeiNa Bio-Henan Provincial Industrial Microbial Strain Engineering and Technology Research Centre, Shangcheng, China). All reagents used in this assay were of chromatographic or analytical grade.

### 2.2 Preparation of SDF

#### 2.2.1 Seed medium preparation

In a sterile environment, a single colony of *Lactiplantibacillus plantarum* BNCC 339790 was cultured by inoculating it into the liquid medium at 37 °C for 20 h. The culture was then transferred at a 5 % ratio to another liquid medium at 37 °C for 20 h (Ma et al., 2022).

#### 2.2.2 Mixed fermentation

The skimmed PS was blended with distilled water at a proportion of 1:95, sterilized at 121 °C, inoculated with 9 % seed medium into sterilized PS, and incubated at 37 °C for 48 h.

#### 2.2.3 SDF extraction

Upon completion of fermentation, the pH was changed to 6–7 using appropriate acids or bases, followed by the incorporation of high-temperature-resistant α-amylase and treated at 95 °C for 35 min to achieve the initial decomposition of starch. When cooled to 55 °C, neutral protease was incorporated and maintained at 55 °C for 30 min to disintegrate proteins further. The pH was adjusted to 4.5, followed by the incorporation of saccharase and treatment for 30 min at 60 °C to complete the saccharification reaction. After centrifugation (5000 r/min, 15 min), the supernatant was concentrated to reduce its volume, precipitated with ethanol, and centrifuged (5000 r/min, 15 min) again to collect the precipitate, which was then lyophilized to obtain SDF (Jiao et al., 2025).

### 2.3 Preparation of modified SDF

#### 2.3.1 Ultrasonic treatment of SDF

The method was adapted from Jiang et al. (2024), with modifications for improvement. Two grams of SDF extracted in the previous section was taken and placed in a 500 mL beaker, distilled water was added, and the mixture was prepared at a ratio of 1:100. The solution was treated for 30 min under a power of 500 W using an ultrasonic cell crusher equipped with a probe system (Shanghai Biron Instrument Manufacturing Co., Ltd., Shanghai, China). It was precipitated with ethanol, centrifuged (5000 r/min, 15 min), and the supernatant was discarded to obtain ultrasonic-treated SDF (U-SDF).

#### 2.3.2 Microwave treatment of SDF

The method was adapted from Jiang et al. (2024), with modifications for improvement. Two grams of SDF extracted in the previous section was taken and placed in a 500 mL beaker, and distilled water was added in a proportion of 1:100, followed by thorough mixing. The mixture was treated at 500 W for 5 min using a microwave chemical reactor (Nanjing Xian'ou Instrument Manufacturing Co., Ltd., Nanjing, China). After cooling, it was precipitated with ethanol, centrifuged (5000 r/min, 15 min), and the supernatant was discarded to obtain microwave-treated SDF (M-SDF).

#### 2.3.3 Ultra-high-pressure treatment of SDF

The method was adapted from Sang et al. (2022), with modifications for improvement. Two grams of SDF extracted in the previous section was taken and placed in a a 10 cm × 15 cm nylon vacuum bag, and distilled water was added in a proportion of 1:100, the nylon bag is sealed, followed by thorough mixing. The mixture was treated for 15 min under a pressure of 400 MPa using an ultra-high-pressure equipment (Shanxi Leadfoam Technology Co., Ltd., Taiyuan, China). After treatment, it was precipitated with ethanol, centrifuged (5000 r/min, 15 min), and the supernatant was discarded to obtain ultra-high-pressure-treated SDF (UHP-SDF).

### 2.4 Determination of basic physicochemical properties

#### 2.4.1 Determination of monosaccharide composition

The sample was placed in an acid digestion bottle, and a hydrochloric acid-methanol solution was added. After filling with N₂, the bottle was quickly sealed and hydrolyzed at 80 °C for 12 h, followed by blow-drying under vacuum. TFA solution was then added, and the mixture was hydrolyzed in a 120 °C constant-temperature metal bath for 1 h before being blown dry again. NaOH and an equal volume of PMP-methanol solution were added, mixed thoroughly, and incubated in a 70 °C water bath for 30 min. HCl solution and chloroform were added; after vigorous shaking, the chloroform layer was discarded to retain the aqueous layer, and this extraction process was repeated three times. The final product was filtered through a 0.22 μm membrane and analyzed by HPLC (Shimadzu Corporation, Tokyo, Japan) using a DIKMA Inertsil ODS-3 column (4.6 × 150 mm). The mobile phase, consisting of PBS (0.1 mol/L, pH 7.0) and acetonitrile (82:18, V/V), was delivered at a flow rate of 1 mL/min. A 20 μL aliquot was injected, and detection was performed at 245 nm (Yu et al., 2023).

#### 2.4.2 Determination of water holding capacity (WHC)

SDF before and after modification (0.1 g) was weighed, mixed with 10 mL of distilled water, and vibrated at 37 °C for 60 min. The mixture was centrifuged at 5000 r/min for 20 min, the supernatant was discarded, and the wet weight of the water-absorbed SDF was measured (Xia et al., 2018). The WHC is computed based on Eq.1.

(1) $$\mathrm{WHC}\;\left(g/g\right)=\frac{m_{1}‐m_{2}}{m_{2}}$$

where, m_1_ is the wet gravity of the sample after water absorption; m_2_ is the dry gravity of the sample.

#### 2.4.3 Determination of oil holding capacity (OHC)

SDF before and after modification (0.1 g) was weighed, mixed with 10 mL of edible oil, and vibrated at 37 °C for 60 min. The mixture was centrifuged at 5000 r/min for 20 min, the supernatant was discarded, and the wet weight of the oil-absorbed SDF was measured (Xia et al., 2018). The OHC is computed based on Eq.2.

(2) $$\mathrm{OHC}\;\left(g/g\right)=\frac{m_{1}‐m_{2}}{m_{2}}$$

where, m_1_ is the wet gravity of the sample after oil absorption; m_2_ is the dry gravity of the sample.

### 2.5 Structural characterization

#### 2.5.1 Fourier infrared spectroscopy (FT-IR)

Unmodified and modified SDF samples were separately mixed with KBr at a ratio of 2:200 (w/w). Each mixture was pressed into pellets and analyzed by FT-IR spectroscopy (Thermo Fisher Scientific, Waltham, USA) across the 400–4000 cm^−1^ range, using pure KBr as the background reference.

#### 2.5.2 X-ray diffraction (XRD)

The crystallinity of SDF was analyzed using an XRD (Dandong Tongda Technology Co., Ltd., Dandong, China). The samples were performed at 30 kV and 10 mA, with the 2θ angle scanned from 5° to 90° at 5°/min.

#### 2.5.3 Thermal stability measurement

The thermal stability of unmodified and modified SDF samples was evaluated using a thermogravimetric analyzer (Netzsch, Selb, Germany) following established methods with modifications (Tan et al., 2024). Five milligrams SDF was positioned in a porcelain crucible with a lid, and high-purity N_2_ was utilized as the carrier gas. The heating rate was set at 20 °C/min, the temperature range was from 35 to 600 °C, and the N₂ flow rate was maintained at 50 mL/min.

#### 2.5.4 Scanning electron microscopy (SEM)

The surface morphology of SDF was assessed by SEM (Shimadzu Corporation, Tokyo, Japan). A few SDFs were distributed uniformly on a conductive adhesive sample stage and sprayed with gold under vacuum. They were photographed at 2000× magnification to investigate the surface and microscopic characteristics of the SDFs (Li et al., 2024).

### 2.6 In vitro hypolipidemic activity studies

#### 2.6.1 Determination of cholesterol adsorption capacity (CAC)

One gram of SDF was mixed with 50 mL of egg yolk solution (egg yolk: distilled water = 1:1), and the system's pH was adjusted to 2 and 7, and the reaction was vibrated at 37 °C for 120 min. After centrifugation (5000 r/min, 15 min), the supernatant (0.1 mL) was transferred to a cuvette, and glacial acetic acid was used to adjust the volume to 4 mL. Two milliliters of o-phthalaldehyde solution was added, followed by the addition of 4 mL of mixed acid (H_2_SO_4_:glacial acetic acid = 1:1) after 5 min; the absorbance was recorded at 550 nm after 15 min (Si et al., 2023). The CAC was computed in line with Eq.3.

(3) $$\mathrm{CAC}\;\left(\mathrm{mg}/g\right)=\frac{\left(m_{2}‐m_{1}\right)}{w}$$

where, m_1_ and m_2_ respectively stand for the cholesterol mass present in the solution prior to and subsequent to adsorption, w denotes the mass of the sample.

#### 2.6.2 Determination of the ability to inhibit pancreatic lipase activity

The method was based on published literature methods with suitable adaptations (Liu et al., 2022). One hundred microliters of SDF solution before and after modification with different concentration gradients were mixed with equivalent amounts of pancreatic lipase solution and Tris-HCl solution at 37 °C for 10 min, and 100 μL of PNPP was added and reacted at 37 °C for 20 min. The mixture was heated at 100 °C for 5 min, and the absorbance was recorded at 540 nm. The inhibition ability of SDF on pancreatic lipase activity was computed in line with Eq.4.

(4) $$\text{Inhibition Rate}\;\left(\%\right)=\left(1‐\frac{A_{4}‐A_{3}}{A_{2}‐A_{1}}\right)\times 100\%$$

where, A_4_ is the sample group; A_3_ is the sample control group; A_2_ is the blank group; and A_1_ is the empty control group.

### 2.7 In vitro hypoglycemic activity studies

#### 2.7.1 Determination of glucose adsorption capacity (GAC)

Based on published literature methods with suitable modifications. Mix 0.1 g of SDF before and after modification with 5 mL glucose solution (0.5 mg/mL) and shake at 37 °C for 120 min. After centrifugation (5000 r/min, 15 min), the supernatant (1 mL) was transferred to a cuvette and mixed well with 1 mL of distilled water and a diploid product of DNS. The mixture was heated at 100 °C for 6 min, and the absorbance was recorded at 540 nm (Yang et al., 2022). The GAC of SDF was computed in line with Eq.5.

(5) $$\mathrm{GAC}\;\left(\mathrm{mg}/g\right)=\frac{\left(m_{2}‐m_{1}\right)}{w}$$

where, m_1_ and m_2_ are the mass of glucose in solution before and after adsorption, w is the mass of the sample.

#### 2.7.2 Determination of inhibitory ability to α-amylase activity

One hundred microliters of SDF solution with different concentration gradients were mixed with an equal volume of α-amylase solution (4 U/mL) and incubated at 37 °C for 10 min. Two hundred microliters of 1 % starch solution was added to the mixture, followed by a 10 min reaction at 37 °C. Adding 4 mL of DNS solution was followed by heating the mixture at 100 °C for 5 min, and the absorbance was recorded at 540 nm (Xiao et al., 2024). The inhibitory ability of SDF on α-amylase activity was computed in line with Eq.6.

(6) $$\text{Inhibition Rate}\;\left(\%\right)=\left(1‐\frac{A_{4}‐A_{3}}{A_{2}‐A_{1}}\right)\times 100\%$$

where, A_4_ is the sample group; A_3_ is the sample control group; A_2_ is the blank group; and A_1_ is the empty control group.

#### 2.7.3 Determination of inhibitory ability on α-glucosidase activity

SDF solution before and after modification (50 μL) at different concentration gradients were mixed with a diploid product of α-glucosidase solution (0.1 U/mL), and the reaction proceeded at 37 °C for 10 min. PNPG solution (50 μL) was introduced and reacted at 37 °C for 10 min. A sodium carbonate solution was used to stop the reaction, and the absorbance was recorded at 405 nm (Yu et al., 2025). The inhibitory ability of SDF on α-glucosidase activity was computed in line with Eq.7.

(7) $$\text{Inhibition Rate}\;\left(\%\right)=\left(1‐\frac{A_{4}‐A_{3}}{A_{2}‐A_{1}}\right)\times 100\%$$

where, A_4_ is the sample group; A_3_ is the sample control group; A_2_ is the blank group; and A_1_ is the empty control group.

### 2.8 In vitro evaluation of immunological activity

#### 2.8.1 Experiment of cytotoxicity

After reaching the logarithmic growth phase, RAW264.7 macrophages were digested with trypsin, and the cell concentration was adjusted accordingly, inoculated in 96-well plates at 6000 cells/well, and cultivated for 24 h. After wall attachment, 50, 100, and 200 μg/mL of SDF before and after the modification and 1 μg/mL of LPS were introduced into the wells, and the medium blank was used as a blank control. After 24 h, the supernatant was aspirated, and 100 uL of 10 % CCK8 solution was injected into the wells and cultivated for 2 h, and the absorbance was measured at 450 nm. Cell viability was computed in line with Eq.8.

(8) $$\text{Cell Viability}\;\left(\%\right)=\frac{A_{1}‐A_{0}}{A_{2}‐A_{0}}\times 100\%$$

where, A_1_ is the sample group; A_2_ is the sample control group; A_0_ is the blank group.

#### 2.8.2 Determination of NO and cellular inflammatory factors

After reaching the logarithmic growth phase, RAW264.7 macrophages were digested with trypsin, and the cell concentration was adjusted accordingly, inoculated in 96-well plates at 6000 cells/well, and cultivated for 24 h. After wall attachment, 50, 100, and 200 μg/mL of SDF before and after the modification and 1 μg/mL of LPS were introduced into the wells, and the medium blank was used as a blank control.

After 24 h, the NO_2_^−^ level in cell supernatant was detected by Griess reaction using a NO detection kit. According to the kit's instructions, 50 μL of cell culture supernatant was added to the enzyme labeling plate, and the absorbance value was measured at 540 nm. The standard curve was plotted according to the standard.

After 24 h, the TNF-α, IL-6, and IL-1β content in the cell supernatant was determined by solid-phase sandwich enzyme-linked immunosorbent assay (ELISA) using the ELISA kit. According to the kit's instructions, 50 μL of cell culture supernatant was added to the enzyme plate, and the absorbance was measured at 450 nm. The standard curve was plotted according to the standards.

### 2.9 Statistical analysis

All experiments were conducted in triplicate. The results are expressed as the mean ± standard deviation (SD). The experimental data were plotted and graphed using Origin 2022 (OriginLab Corporation, Northampton, MA, USA), and the software IBM SPSS Statistics 27 (IBM Corporation, Armonk, NY, USA) was used for statistical analysis of the experimental data. The experimental data were analyzed using one-way Analysis of Variance (ANOVA). Statistical differences were considered significant at *p < 0.05*.

## 3 Results and discussion

### 3.1 Analysis of physical and chemical properties

#### 3.1.1 Analysis of monosaccharide composition

As shown in Table 1, the main components of SDF before and after modification is composed primarily of galactose, glucose, galacturonic acid, and arabinose, which are in agreement with the results of Wang et al. (2023). Compared with unmodified SDF, the amount of glucuronic acid, mannose, galacturonic acid, and galactose in the modified SDF increased. The ultrasonically induced cavitation and mechanical vibration may cause the molecular chain of SDF to be broken at certain positions, and the reorganization of the molecular chain after the break exposes more carboxyl groups, resulting in higher levels of glucuronic acid and galacturonic acid (Gao et al., 2025). The local high temperature and rapid molecular movement of microwaves, while less intense than that of ultrasound, also induce the breakage and recombination of some chemical bonds, which consequently affect the relevant structure of monosaccharides, and the thermal effect of microwaves may foster the oxidation of the hydroxyl group on the polysaccharide chain to the aldehyde group, which is further transformed into the carboxyl group. Ultra-high-pressure treatment, on the other hand, affects the structural regions of monosaccharides by altering the conformation and hydrogen bonding network of SDF molecules, favoring the presentation of mannose. In addition, ultra-high-pressure might modify the intermolecular interactions and the stability of chemical bonds and facilitate the formation and exposure of relevant functional groups. This finding is in line with the research outcomes of Zheng et al. (2024). In conclusion, the different modification approaches did not alter the types of monosaccharides in SDF but had varying effects on their molar fractions.

**Table 1:** Effects of different modification methods on the monosaccharide composition of SDF.

	**SDF**	**U-SDF**	**M-SDF**	**UHP-SDF**
Mannose (mol%)	5.75 ± 0.03^d^	7.35 ± 0.03^b^	7.25 ± 0.05^c^	8.59 ± 0.04^a^
Glucuronic acid (mol%)	1.41 ± 0.02^d^	2.04 ± 0.04^c^	2.29 ± 0.02^a^	2.12 ± 0.05^b^
Rhamnose (mol%)	8.08 ± 0.05^a^	6.23 ± 0.04^c^	6.41 ± 0.03^b^	5.7 ± 0.07^d^
Galacturonic acid (mol%)	22.17 ± 0.02^d^	32.62 ± 0.03^b^	31.71 ± 0.05^c^	33.11 ± 0.04^a^
Glucose (mol%)	14.74 ± 0.04^a^	8.98 ± 0.03^c^	9.06 ± 0.05^b^	8.74 ± 0.05^d^
Galactose (mol%)	23.82 ± 0.05^d^	24.71 ± 0.04^b^	24.23 ± 0.05^c^	25.51 ± 007^a^
Xylose (mol%)	3.71 ± 0.05^a^	3.06 ± 0.03^c^	3.11 ± 0.03^b^	2.62 ± 0.02^d^
Arabinose (mol%)	19.03 ± 0.03^a^	14.39 ± 0.03^c^	15.26 ± 0.03^b^	13.16 ± 0.05^d^
Fucose (mol%)	1.28 ± 0.03^a^	0.63 ± 0.01^c^	0.67 ± 0.01^b^	0.45 ± 0.04^d^

Note: Different letters indicate significant differences between the data (*p ˂ 0.05*).

#### 3.1.2 WHC and OHC analysis

Due to its superior WHC and OHC, SDF is highly suitable for various food industry applications, including a thickener, stabilizer, or ingredient in functional food formulations. As presented in Table 2, the WHC and OHC of U-SDF, M-SDF, and UHP-SDF were significantly enhanced compared to unmodified SDF (*p < 0.05*). The modification treatment may disrupt the macromolecular structure of SDF, giving rise to a more relaxed and porous configuration. This process may expose the hydrophilic and hydrophobic groups in SDF, thus strengthening its adsorption capacity for water and oil, it also facilitates the rupture and rearrangement of molecular chains, which may modify the hydrogen bonding and other intermolecular forces within and between SDF molecules, and the new distribution of forces may enable SDF to create a spatial structure more favorable to water and oil retention, such as the formation of microcapsule-like structures in which water or oil is encapsulated (Li et al., 2022). Therefore, it can be deduced that appropriate modification treatments can improve the WHC, OHC, and bioactivity of SDF from PS.

**Table 2:** Effect of different modification methods on WHC and OHC of SDF.

	**SDF**	**U-SDF**	**M-SDF**	**UHP-SDF**
WHC (g/g)	2.53 ± 0.25^c^	4.07 ± 0.15^a^	3.67 ± 0.15^b^	4.40 ± 0.20^a^
OHC (g/g)	6.33 ± 0.15^c^	11.47 ± 0.15^b^	11.70 ± 0.20^b^	13.13 ± 0.21^a^

Note: Different letters indicate significant differences between the data (*p ˂ 0.05*).

### 3.2 Structural analysis

#### 3.2.1 FT-IR analysis

**Fig. 1:**
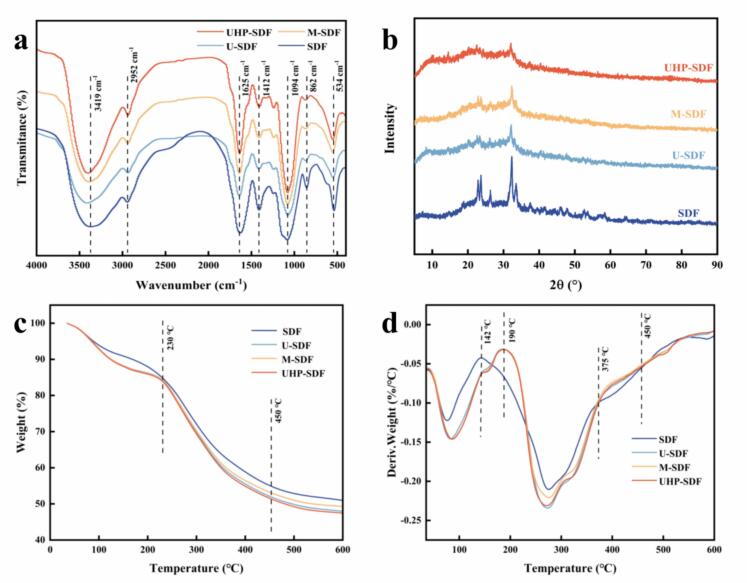
Effect of different modification methods on the structure of SDF. (a) FT-IR of SDF by modification; (b) XRD plots of SDF before and after modification; (c) TG plots of SDF before and after modification; (d) DTG plots of SDF before and after modification.

Note: SDF: unmodified; U-SDF: obtained by ultrasonic modification; M-SDF: obtained by microwave modification; UHP-SDF: obtained by ultra-high-pressure modification.

Fig. 1a presents the infrared spectra of SDF of PS before and after modification, the curves of SDF before and after modification showed different trends in different wave number ranges, indicating that the modification affected the structure of SDF, thus affecting the infrared absorption characteristics. The vast peaks of 3200–3600 cm^−1^ may correspond to the stretching vibrations of hydroxyl groups, suggesting that the four SDF samples are rich in hydroxyl groups, such as those found in cellulose, hemicellulose, and pectin, and that the peaks before and after modification are slightly different in their positions and shapes (Niu et al., 2018). The peak around 2930 cm^−1^ is attributed to the C—H stretching vibration of the methyl and methylene group. 1630 cm^−1^ may be the telescopic vibrational peak of the carbonyl group in the free carboxyl group. The peak intensity is associated with pectin content, and it can be concluded that the content of free carboxyl groups is increased in M-SDF and UHP-SDF (Dong et al., 2022). The peak around 1420 cm^−1^ indicates that the lignin causes the aromatic or aliphatic C—H stretching vibration, and it suggests that the content of lignin in the modified SDFs is reduced compared with that of the unmodified ones. In addition, the peak around 1100 cm^−1^ is related to the C—O stretching vibration in lignin and the stretching vibration of the acyloxy group in hemicellulose, and it can be concluded that the lignin and hemicellulose contents of U-SDF are the lowest. 865 cm^−1^ is generated by the stretching vibration of the β-glycosidic bond, and the modification treatment may change the absorption peaks composed of glucose, galactose, mannose, and other monosaccharides connected by β-glycosidic bonds, which are responsible for the changes in monosaccharide composition. The results showed that none of the peak patterns of the modified SDF changed, and there were only changes in the intensity of specific peaks, indicating that no new substances were produced, but only some chemical groups were enhanced.

#### 3.2.2 XRD analysis

Fig. 1b presents the XRD patterns of SDF before and after modification. SDF has several prominent diffraction peaks, and there is a pronounced diffraction peak with high intensity at 2θ about 20°, which reflects the specific crystal arrangement and ordering in its crystal structure. In addition, there are diffraction peaks with varying intensities at positions such as 2θ about 30°-35°, which suggests that it has a specific crystalline structure. The position and intensity of the peaks of U-SDF were changed, and the intensity of the diffraction peak at 20° was weakened, while a relatively prominent new peak appeared at 32°. This indicates that the ultrasonic modification has caused significant changes to the crystal structure of SDF, the diffraction peaks at other angles are also significantly different from that of SDF, and a new structure may have been formed. This finding aligns with the results reported by Qiao et al. (2021) research regarding sweet potato residue. M-SDF has a diffraction peak at 23°, which is different from that of SDF, and there is also a difference in the peak intensity, in the range of 30°-35°, the pattern of the diffraction peaks is different from that of both SDF and U-SDF, indicating that the microwave treatment has altered the crystalline properties of the SDF, which may result in the change of the lattice parameter or the change of the crystalline orientation. The degree of damage to the crystal structure of SDF by ultra-high-pressure modification is relatively small, and SDF still maintains a relatively stable crystal structure after ultra-high-pressure treatment. These differences in the crystal structure will probably result in differences in the properties of SDF, providing a structural level for the selection of suitable modification methods for its application in food, medicine, and other fields.

#### 3.2.3 Thermal stability analysis

Fig. 1c and Fig. 1d illustrate the pyrolysis processes of SDF before and after modification from 35 °C to 600 °C, analyzing the influence that the various modification methods devised have on the thermal stability of SDF. In the starting stage (35–200 °C), all four samples showed a slight weight loss, probably caused by the dissipation of volatile substances such as moisture adsorbed in the samples (Dong et al., 2020). The weightlessness rate of SDF was relatively high, and the weight loss rates of U-SDF, M-SDF, and UHP-SDF were relatively low and close to each other, indicating that ultrasonic, microwave, and ultra-high-pressure treatments may have altered the state of moisture presence in the samples to some extent. In the second stage (200–450 °C), the polysaccharide chain breaks as the temperature increases, causing weight loss of the samples. SDF has a faster rate of weight loss in this stage, with a more significant weight loss from about 90 % to about 50 %. The weightlessness rate of the samples with the three modification treatments was slightly slower. The weight loss amplitude was relatively small compared to that of SDF, suggesting that ultrasonication, microwave, and ultra-high-pressure treatments enhanced the thermal stability of the samples, which may have altered the structure of SDF to make it more stable in the thermal decomposition process. In the last stage (450–600 °C), the weightlessness rate of the four samples was significantly slowed down, the weight loss amplitude was smaller, the SDF was bio‑carbonated, and the pyrolysis rate inclined to stabilize. Overall, the thermal stability of both U-SDF and UHP-SDF was lower than that of M-SDF and SDF.

#### 3.2.4 SEM analysis

**Fig. 2:**
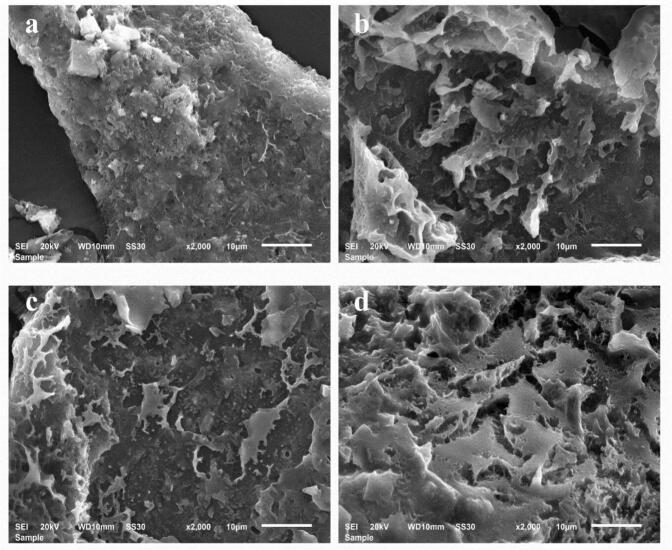
SEM images of SDF before and after modification. (a) Image of SDF at 2000× magnification; (b) Image of U-SDF at 2000× magnification; (c) Image of M-SDF at 2000× magnification; (d) Image of UHP-SDF at 2000× magnification.

SEM is an essential tool to study the microstructure of polysaccharide samples. The microstructures of SDF are shown in Fig. 2, where a, b, c and d show the microstructures of SDF, U-SDF, M-SDF and UHP-SDF at a magnification of 2000×, respectively. Fig. 2a demonstrates the unmodified SDF, which features a relatively smooth surface and a tight structure. Showing a denser state indicates that the intermolecular interactions between the unmodified treated SDF molecules are tight and well-arranged. Fig. 2b shows the ultrasonically modified U-SDF with irregular pore morphology in the microstructure and a further increase in the looseness of the structure. This is comparable to the outcomes of Gao et al. (2025) in the study of mycelial polysaccharides from Sanghuang, which explains that the ultrasonication treatment destroys some of the intermolecular forces between SDF molecules, which makes the structure looser and creates more porous structures. Fig. 2c shows the microwave-modified M-SDF, whose surface becomes significantly rougher, and more pores appear than the unmodified SDF. The chemical bonds and interaction forces between molecules are broken and reorganized during the microwave treatment, forming this more loose and porous structure. Fig. 2d presents the UHP-SDF modified by ultra-high-pressure, the microstructure of which shows more abundant pores and a looser overall structure. These structural features of the modified SDF affected its physicochemical properties; at the same time, the rough structure may have a role in the adsorption capacity of cholesterol and glucose, which could adsorb cholesterol in the intestines more efficiently and reduce its absorption into the bloodstream. Through these mechanisms of action, the modified SDF shows the potential efficacy of hypoglycemic and hypolipidemic effects, which has a broad application prospect in functional foods and related health fields.

### 3.3 Analysis of in vitro hypolipidemic capacity

#### 3.3.1 CAC analysis

**Fig. 3:**
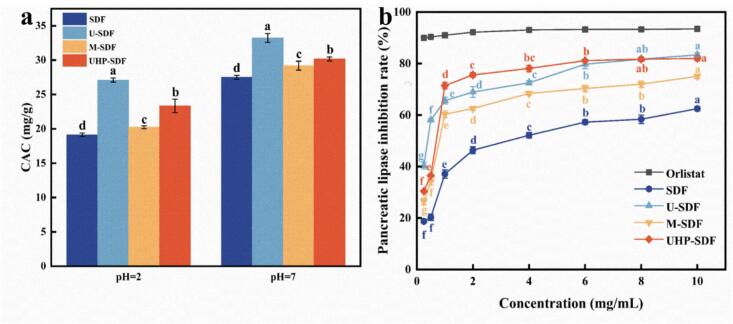
Plots of in vitro lipid-lowering ability analysis of SDF before and after modification. (a) Effect of modification on CAC of SDF; (b) Effect of modification on the inhibitory ability of pancreatic lipase activity of SDF. Note: Different letters indicate significant differences between the data (*p ˂ 0.05*).

The CAC of SDF is a crucial functional feature, as SDF facilitates cholesterol adsorption through physical adsorption and gel formation. Fig. 3a depicts the CAC of SDF. The CAC of SDF showed a clear environmental dependence, with significantly higher efficacy under intestinal conditions (pH 7) than gastrointestinal conditions (pH 2) (*p < 0.05*), SDF's carboxyl groups (-COOH) deprotonate at neutral pH, enhancing electrostatic interactions with cholesterol. At pH 2: SDF (19.14 ± 0.25 mg/g) < M-SDF (20.23 ± 0.21 mg/g) < UHP-SDF (23.33 ± 0.97 mg/g) < U-SDF (27.1 ± 0.30 mg/g). At pH 7: SDF (27.5 ± 0.30 mg/g) < UHP-SDF (30.17 ± 0.31 mg/g) < M-SDF (29.2 ± 0.66 mg/g) < U-SDF (33.23 ± 0.65 mg/g). Results indicated that the CACs of SDFs modified by ultrasound, microwave, and ultra-high-pressure were higher than those of the unmodified ones, which might be associated with their rough surface and larger specific surface area. Yang et al. (2024) investigated the influence of diverse methods of extraction (e.g., hot water treatment, ultrasonic treatment) on the structural and functional properties of sweet potato SDF. The ultrasound-assisted extracted SDF had better CAC than other extraction methods, which is consistent with the results of this study.

#### 3.3.2 Analysis of the inhibitory ability of pancreatic lipase activity

SDF forms a gel-like substance in the intestines, which increases the viscosity of intestinal contents and prevents the interaction between pancreatic lipase and the substrate, thereby lessening the digestion of fats. As depicted in Fig. 3b, SDF's pancreatic lipase inhibitory activity before and after modification tended to rise with the sample concentration (*p < 0.05*). The increase in inhibition rate was more evident at lower concentrations, and when the concentration reached a certain level, the rise in inhibition rate tended to level off. The pancreatic lipase inhibition rates of SDF, U-SDF, M-SDF, and UHP-SDF were 62.44 ± 0.84 %, 83.33 ± 0.84 %, 75.00 ± 0.83 %, and 81.94 ± 0.96 %, respectively, at sample concentrations up to 10 mg/mL. The IC_50_ of SDF, U-SDF, M-SDF, and UHP-SDF was 3.300, 0.372, 1.087 and 0.643 mg/mL, respectively. According to the IC_50_ data, several samples' pancreatic lipase inhibition ability was U-SDF > UHP-SDF > M-SDF > SDF, demonstrating that ultrasound treatment effectively increased the pancreatic lipase suppression ability. However, the overall inhibition ability was lower than that of orlistat. The increased pancreatic lipase inhibition of modified SDF might play a role in weight control and prevent obesity and related metabolic diseases. It may also regulate intestinal flora, improve intestinal microenvironment, and promote overall health (Ding et al., 2020).

### 3.4 Analysis of in vitro hypoglycemic capacity

#### 3.4.1 GAC analysis

**Fig. 4:**
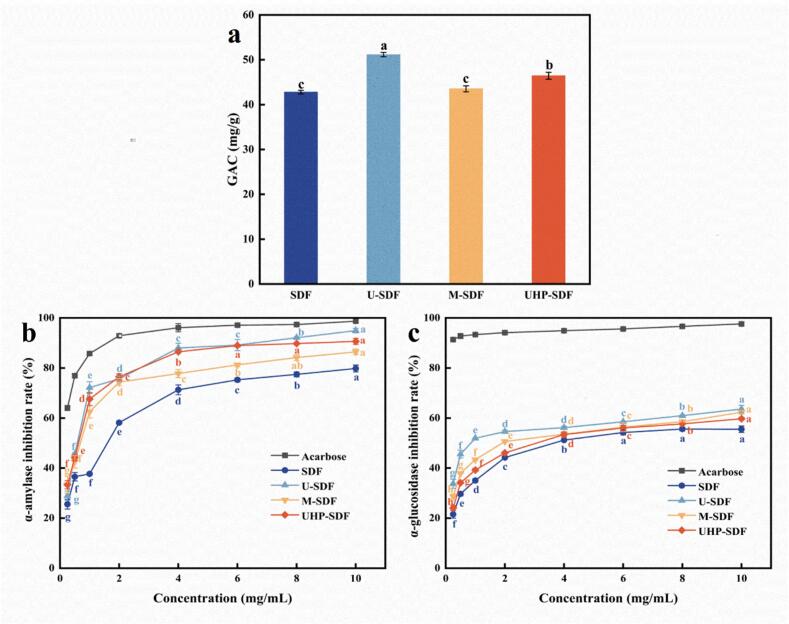
Plots of in vitro hypoglycemic capacity analysis of SDF before and after modification. (a) effect of modification on GAC of SDF; (b) effect of modification on inhibitory ability of α-amylase activity of SDF; (c) effect of modification on inhibitory ability of α-glucosidase activity of SDF. Note: Different letters indicate significant differences between the data (*p ˂ 0.05*).

GAC serves as a key indicator for assessing the functional properties of SDF. SDF molecules include many hydrophilic groups interacting with glucose molecules and adsorb glucose onto their surface using their chemical structure. Since the GAC of SDF is beneficial for blood glucose control, it is widely employed in developing functional foods. The GAC of SDF before and after modification is shown in Fig. 4a. The GAC of SDF, U-SDF, M-SDF, and UHP-SDF were 42.80 ± 0.40, 51.17 ± 0.47, 43.53 ± 0.67, and 46.63 ± 0.78 mg/g, respectively. The GAC of U-SDF was the highest, as ultrasonic treatment destroys the intermolecular forces of SDF, leading to a loose and disordered structure, which increases the specific surface area, providing more adsorption sites for glucose. Meanwhile, the hydrophilic groups originally wrapped in SDF are more exposed, and form hydrogen bonds with glucose molecules and have other interactions, thus enhancing the adsorption capacity. Dhar and Deka (2023) indicated a remarkable growth in GAC of DF extracted from pineapple wastes by the ultrasound-assisted method. The modified SDF embodied significant glucose adsorption capacity, which helps delay glucose absorption from the intestines, decreasing postprandial blood glucose levels. This is essential for preventing and controlling metabolic diseases such as diabetes.

#### 3.4.2 Analysis of α-amylase activity inhibition ability

The pronounced viscosity and web structure of SDF increase the viscosity of the gastrointestinal tract contents, which hinders the access of α-amylase to starch and further reduces the enzyme activity, and also influences glucose diffusion and absorption in the intestinal tract, which serves to smooth out the blood glucose. As depicted in Fig. 4b, the α-amylase inhibitory activity of SDF before and after modification showed an increasing trend with increasing sample concentration (*p < 0.05*). When the sample concentration reached 10 mg/mL, the α-amylase inhibition of SDF, U-SDF, M-SDF, and UHP-SDF were 79.81 ± 1.39 %, 94.94 ± 0.87 %, 86.42 ± 1.09 %, and 90.64 ± 1.17 %, respectively. The IC_50_ of SDF, U-SDF, M-SDF, and UHP-SDF were 1.307, 0.556, 0.621, and 0.541 mg/mL, respectively. From the IC_50_ data, the inhibition ability of several samples to inhibit α-amylase was in the order of strongest to weakest as UHP-SDF > U-SDF > M-SDF > SDF, which can be obtained that the inhibition ability of UHP-SDF and U-SDF on α-amylase activity was significantly improved, thus achieving the purpose of hypoglycemia. Wang et al. (2024) extracted SDF from the calyx of sour pulp by ultrasound-assisted method, and the results showed that it had a good inhibition of α-amylase. The IC_50_ reached 1.2 mg/mL, and our results of the present study were better than his effect. By inhibiting α-amylase activity, the modified SDF inhibited the rapid decomposition of starch in the intestine, thus delaying the release and absorption of glucose and helping to reduce the postprandial blood glucose peak. Long-term intake of SDF-rich foods can improve glucose metabolism and reduce the incidence of type II diabetes.

#### 3.4.3 Analysis of α-glucosidase activity inhibition ability

SDF's suppressive influence on α-glucosidase is a crucial mechanism for its hypoglycemic function. The structure of SDF is analogous to the active site of α-glucosidase. When the binding site is occupied, the substrate (oligosaccharide, disaccharide, etc.) cannot be bound usually, so the enzyme cannot catalyze the hydrolysis of the substrate, suppress the activity of α-glucosidase, and decrease the release of glucose (Xiong et al., 2022). At the same time, the high molecular weight or viscosity of SDF may form a physical barrier that hinders enzyme-substrate contact and reduces enzymatic efficiency. As seen in Fig. 4c, the α-glucosidase inhibitory activity of SDF before and after modification showed an upward tendency with increasing sample concentration (*p < 0.05*). The α-glucosidase inhibition of SDF, U-SDF, M-SDF, and UHP-SDF were 55.52 ± 1.30 %, 63.57 ± 1.50 %, 62.30 ± 0.73 %, and 59.70 ± 0.49 %, respectively, at sample concentrations up to 10 mg/mL. The IC_50_ of SDF, U-SDF, M-SDF, and UHP-SDF were 4.317, 1.362, 2.494, and 3.226 mg/mL, respectively. From the IC_50_ data, the inhibitory ability of several samples to inhibit α-glucosidase was in the order of strongest to weakest, with U-SDF > UHP-SDF > M-SDF > SDF and the inhibitory ability of U-SDF to inhibit α-glucosidase was significant. However, its inhibitory effect was always less than that of the control acarbose. Ji et al. (2024) used different extraction methods to extract SDF from blue honeysuckle berries; among them, the SDF extracted by fermentation method showed the highest inhibition of α-glucosidase up to 54.87 ± 1.25 %, and our results of the present study were better than his effect. SDF or its degradation products may affect the spatial conformation of α-glucosidase through non-covalent interactions (e.g., hydrogen bonding, hydrophobic interactions, etc.). Modified SDF has good α-glucosidase inhibition ability and can be added as a food ingredient to make healthy food with a hypoglycemic effect.

### 3.5 In vitro immunological activity analysis

#### 3.5.1 Cell viability analysis

**Fig. 5:**
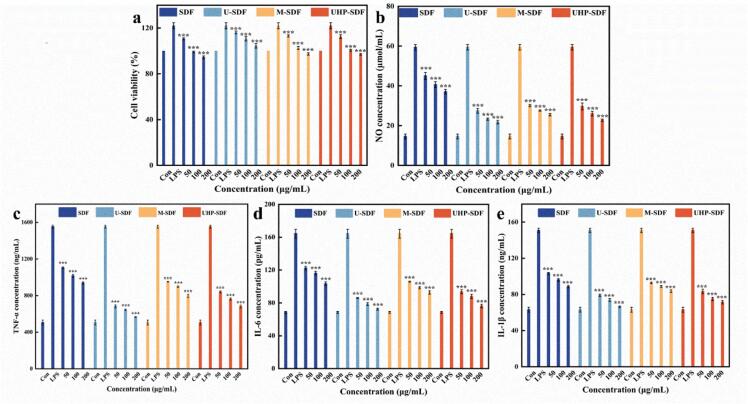
In vitro immunoreactivity analysis graphs of SDF before and after modification. (a) Effect of SDF on the proliferative activity of RAW264.7 macrophages before and after modification; (b) Effect of SDF on NO release from RAW264.7 cells before and after modification; (c) Effect of SDF on secretion of cytokine TNF-α by RAW264.7 cells before and after modification; (d) Effect of SDF on secretion of cytokine IL-6 by RAW264.7 cells before and after modification effect; (e) effect of SDF on secretion of cytokine IL-1β by RAW264.7 cells before and after modification.

Note: *** represents an extremely statistically significant difference (*p < 0.001*).

In this study, the impact of SDF on the viability of macrophage RAW264.7 cells was determined using the CCK8 method. As presented in Fig. 5a, different mass concentrations of SDF had different effects on the viability of RAW264.7 cells, and the cell viability of the improved group was significantly higher than the control group under low concentration conditions (*p < 0.001*). In contrast, the cell viability of the unmodified group was comparable to that of the control group, which showed a promotion effect. This is similar to the discoveries of Rodin et al. (2024) on *Suaeda maritima* polysaccharide attenuating LPS-induced cellular inflammation. With the increase in concentration, the cell viability of all U-SDF groups was greater than that of the blank group, but their viability tended to decrease. The cell viability of the remaining groups decreased at high concentrations, suggesting that a concentration of SDF that is too high may cause some stress or toxicity to the cells. The outcome of this research indicated that the cell viability of RAW264.7 by SDF before and after modification was always higher than 80 % in the concentration range of 50–200 μg/mL, which demonstrated that there was little or no toxic effect on the cells.

#### 3.5.2 Analysis of NO release and cellular inflammatory factors

In cellular anti-inflammatory studies, NO concentration is often used as a key indicator because its concentration change intuitively reflects the degree of activation of the cellular inflammatory response. Fig. 5b shows the effect of SDF on intracellular NO concentration; with the upward trend of the SDF concentration in each group, the concentration of NO showed a decreasing trend, especially at the higher concentration, the concentration of NO in each group was considerably less than that in control, which indicates that the SDF has a particular anti-inflammatory effect, suppress intracellular NO production, thereby reducing the inflammatory response, and shows a concentration dependence. Furthermore, according to the data, the NO concentration of the U-SDF group was at a lower level as a whole, indicating that it had a better anti-inflammatory effect, followed closely by UHP-SDF and M-SDF, and the anti-inflammatory effect of the unmodified SDF group was relatively weak (*p < 0.001*). Ultrasound treatment may have caused changes in the molecular structure of SDF, such as breakage of molecular chains or fuller exposure of functional groups, which enhanced its interaction with cells and inhibited NO production more effectively. This aligns with Peng et al.'s (Peng et al., 2024) anti-inflammatory study of *Armeniaca Sibirica* L. Lam extract, which significantly reduced NO in LPS-stimulated RAW264.7 cells, attenuating the inflammatory response.

Macrophage RAW264.7 releases a wide range of inflammatory factors. To investigate the inflammatory effects of SDF before and after modification, the release of inflammatory factors was quantified with an ELISA kit. The results, as shown in Fig. 5c, Fig. 5d, and Fig. 5e, indicated that with the increase in the concentration of SDF before and after modification, the concentration of TNF-α, IL-6, and IL-1β showed a decreasing trend. The concentration of the inflammatory factors in each group was markedly less than that in the LPS control group (*p < 0.001*). This suggests that both SDF before and after modification can suppress the release of these inflammatory factors, thereby decreasing the inflammatory response (Xiong et al., 2018). At the same concentration, there were differences in the inhibitory effects of U-SDF and UHP-SDF, etc., compared with untreated SDF on TNF-α, IL-6, and IL-1β. For example, the U-SDF group showed relatively more potent inhibition of inflammatory factors at multiple concentrations, suggesting that the ultrasound treatment may have enhanced the anti-inflammatory activity of SDF more effectively. In contrast, there were also different degrees of differences between the other treatment groups, suggesting that different physical treatments altered the structure or properties of SDF, affecting its ability to regulate cellular inflammatory factors. The research results provide an experimental basis for the application of SDF and its modified products of PS in the anti-inflammatory field and provide practical guidance for the development of functional foods or anti-inflammatory drugs based on modified SDF.

## 4 Conclusion

The results indicated that the modification treatments altered the monosaccharide composition of SDF. Additionally, the microstructure of SDF became looser and more porous, and the crystallinity was reduced, which improved the bioavailability of SDF. The WHC, OHC, CAC, and GAC of modified SDF were increased, and its inhibitory effects on α-amylase, α-glucosidase, and pancreatic lipase were improved, which indicated that it had potential applications in hypoglycemic and hypolipidemic experiments. In summary, modification can effectively improve SDF's functional properties and bioactivity. This study conducted a preliminary investigation into the impact of modification on SDF quality, but no in vivo experiments have been conducted. Follow-up research could utilize animal models of diabetes and hyperlipidemia to validate the in vivo efficacy of modified SDF, thereby laying a solid foundation for the subsequent development of functional foods and health supplements.

## CRediT authorship contribution statement

**Qiong Wu:** Resources, Methodology, Conceptualization. **Xinru Wu:** Writing – original draft, Methodology, Investigation. **Zifei Wang:** Writing – original draft, Investigation. **Zhitong Cai:** Writing – original draft. **Yonggang Dai:** Writing – review & editing, Methodology, Conceptualization.

## Funding sources

This study was financially supported by the 10.13039/501100011845College of Food Science and Engineering, Changchun University, Jilin Academy of Agricultural Sciences, and the Jilin Science and Technology Development Program Project (20250203133SF).

## Declaration of competing interest

The authors declare that they have no known competing financial interests or personal relationships that could have appeared to influence the work reported in this paper.

## Data Availability

Data will be made available on request.
